# A Railway Network Dataset Incorporating Multi-Type Train Operation Records and Train Scheduling

**DOI:** 10.1038/s41597-025-06385-8

**Published:** 2025-12-13

**Authors:** Jianqing Wu, Xukai Xiao, Yitao Zhou, Bo Du, Jun Shen, Yishan Chen, Bi Wang, Qiang Wu

**Affiliations:** 1 School of Information Engineering, Jiangxi University of Science and Technology, Ganzhou, Jiangxi Province 341000 China; 2 Department of Management, Griffith University, Brisbane, QLD 4111 Australia; 3 School of Computing and Information Technology, University of Wollongong, Wollongong, NSW 2522 Australia; 4 Collaborative Innovation Center for Western Ecological Safety, Lanzhou University, Lanzhou, China

**Keywords:** Civil engineering, Geography

## Abstract

Train operation data contains valuable information with potential insights, yet the datasets released by railway companies are often unstandardized or incomplete, limiting their direct applicability in research. Publicly available railway network datasets that comprehensively integrate train operation records with scheduling information remain rare. To support research in large-scale complex networks, dynamic systems, and intelligent transportation systems, we present the Italian Railway Network Dataset. This dataset includes operational records of multiple types of trains, station locations, inter-station mileage, weather conditions, and scheduling data. By providing detailed and structured railway data, our dataset facilitates research in diverse areas such as spatio-temporal pattern mining, network topology analysis, and train delay propagation and distribution. Moreover, it offers valuable support for addressing operational challenges in the railway domain, including timetable optimization, system resilience assessment, and advanced scheduling strategies.

## Background & Summary

Rail transportation has evolved into a highly networked and dynamic system, marked by high-speed, high-density, and high-capacity train services^1^. The coordinated operation of multiple train types has become the dominant mode in modern railway networks^2^. In this context, data-driven methodologies have been widely used in intelligent transportation fields, such as train delay prediction and scheduling optimization to improve operational efficiency and service quality^3–6^. Historical train operation data plays a key role in advancing research in intelligent transportation and scheduling. This data includes a wide range of information such as train timetables, station locations, railway trajectories, and records of abnormal events^4,7,8^. These diverse datasets provide a solid foundation for developing accurate and effective train scheduling optimization models. However, several challenges restrict the effective use of such data. Confidentiality concerns, data complexity, and decentralized management often impede their access and applicability^7^. Existing publicly available datasets are often limited in scope. They primarily focus on high-speed railways or specific train types and lack comprehensive coverage of diverse train categories, operational environments, and multi-source heterogeneous data. These limitations restrict the ability to perform cross-modal and multi-dimensional data analysis, thereby hindering advancements in model generalization and the broader applicability of intelligent scheduling systems. Therefore, effectively utilizing available data and exploring multi-source data fusion methods to enhance the accuracy and robustness of train delay prediction and intelligent scheduling optimization has become a critical challenge in intelligent transportation research^9^. The complexity of railway networks is primarily reflected in three key aspects:

1. **Spatio-temporal characteristics of train operations:** Train operations exhibit intricate spatio-temporal dynamics, with varying operational states across both time and space. These dynamics include spatio-temporal distribution patterns, temporal dependencies, and spatial interactions^10–12^. The railway system supports multiple train types, each with distinct spatio-temporal distributions of delays and operational characteristics.
2. **Complex railway network topology:** The topology of railway networks is highly complex due to geographical constraints and economic disparities^13,14^. Infrastructure distribution is often uneven, and certain regions are more susceptible to delays caused by specific geographical and climatic conditions^15^.
3. **Dynamic dependencies in train operations:** Train movements are influenced not only by scheduling rules and network topology but also by external environmental factors such as unexpected incidents, adverse weather, and fluctuating passenger demand^16–18^. Passenger flow varies by time and location, affected by holidays, weekdays, and large-scale events like sports competitions or conferences. These fluctuations can result in speed restrictions or temporary service suspensions.

**Fig. 1:**
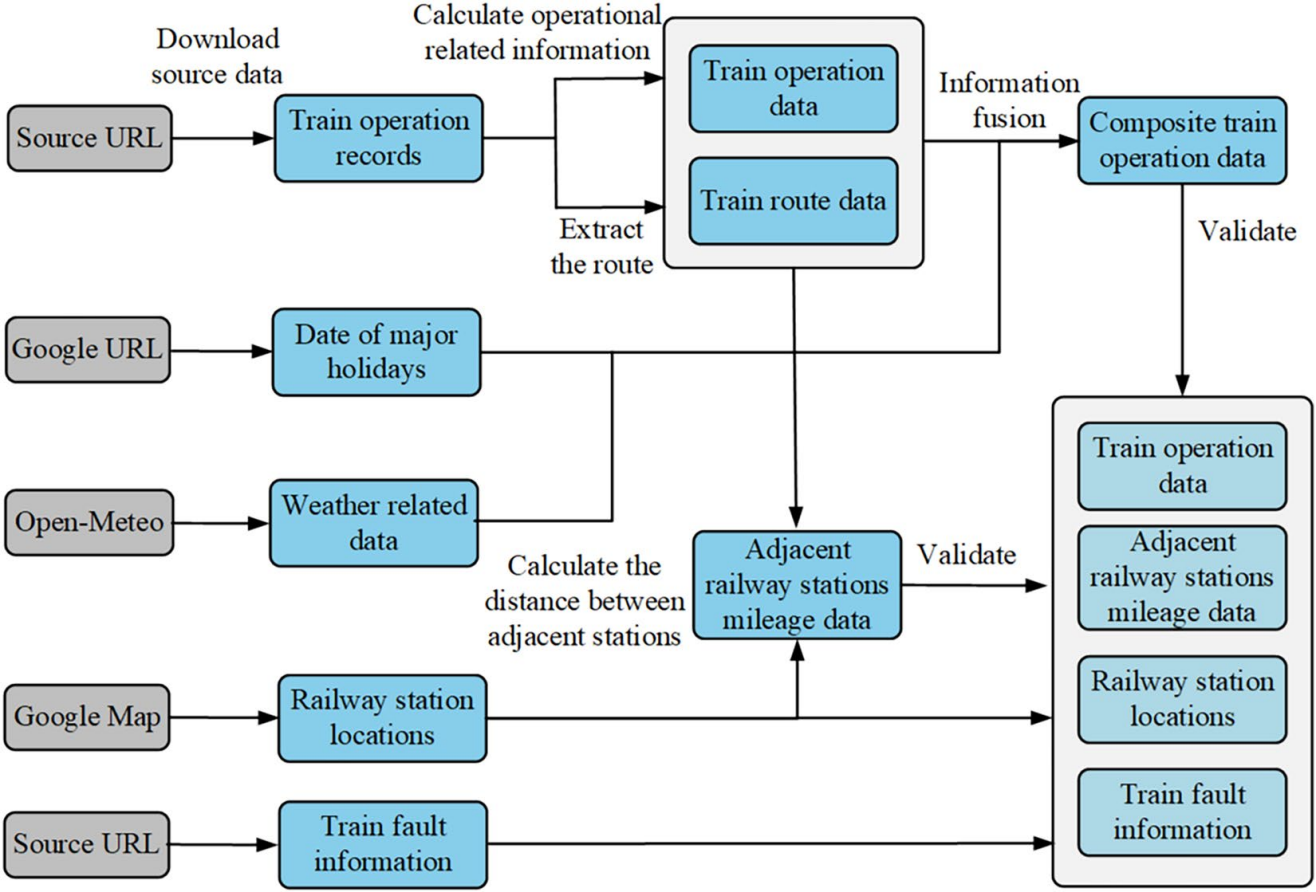
Methodological flowchart for dataset construction.

Figure 1 illustrates the methodological workflow for constructing a railway network dataset based on train operation records and weather data. With the increasing complexity of modern railway systems, efficient coordination among diverse types of trains operating on shared tracks has become a critical challenge. These trains differ significantly in terms of speed profiles, station stopping patterns, operational priorities, and scheduling strategies, contributing to substantial heterogeneity within the network.

To address these challenges, the availability and dissemination of comprehensive datasets that capture multi-category train operation data under real-world conditions are essential. Such datasets not only facilitate the optimization of transportation planning and operational efficiency but also contribute to the development of more effective train scheduling algorithms. Furthermore, they support delay reduction strategies and provide empirical foundations for intelligent transportation systems and automated scheduling decision-making.

In this study, a large-scale and multi-dimensional dataset of the Italian railway network was constructed, encompassing 3,324 trains and 1,397 stations over the period from 1 January to 30 June 2024. The dataset comprises detailed operational records from various train categories, including high-speed, regional, and intercity services. It incorporates geographical attributes of stations, such as geographic coordinates (latitude and longitude) and facility identifiers. Furthermore, the dataset captures network topology and adjacency relationships, including interstation distances and track connectivity.

In addition to structural data, the dataset integrates contextual factors such as weather conditions, national holidays (e.g., New Year’s Day and Easter), and dynamic scheduling adjustments in response to unplanned disruptions, as well as the recorded causes of train delays. These features provide a robust foundation for examining the operational dynamics of the Italian railway system.

From the academic perspective, this dataset constitutes a valuable resource for advancing research in intelligent transportation systems, data mining, complex network modeling, dynamic system behavior analysis, and traffic demand forecasting. Specific applications include train delay analysis^19,20^, railway network topology studies^21^, spatio-temporal pattern recognition^17^, urban accessibility evaluation^22,23^, and anomaly detection in operations^24,25^.

Future research could focus on the development of unified data integration frameworks, the application of deep learning methodologies, and the implementation of knowledge-informed scheduling optimization strategies. From an applied perspective, the dataset offers practical value in supporting train timetable optimization^5,26^, adaptive planning during operational disruptions^27,28^, fault and incident detection^29–31^, and route optimization for railway services^32^. Although the dataset includes high-speed, regional, and intercity trains, other types of trains (e.g., freight trains and special service trains) are not covered. In addition, inter-station distances were calculated using the Haversine formula, which differed from the actual operational distances. To address this limitation, we have provided guidance on obtaining the actual inter-station distances.

## Methods

To construct the Italian railway network dataset, we first downloaded operational records of trains and extracted the geographic locations of railway stations. Subsequently, we calculated the actual arrival and departure times for each train, from which we derived the actual dwell times at stations and the actual travel times between station pairs. Overnight train services were also identified and labeled accordingly. In the third stage, train routes were extracted, and interstation distances were calculated based on the Haversine formula. This was followed by the analysis of operational data specific to major railway hubs across Italy. Additionally, external contextual variables, including station categories, weather conditions, national holidays, and schedule adjustments, were incorporated into the dataset by aligning spatial and temporal metadata, particularly for train operations and station-level delay analysis. Finally, a validation step was conducted to ensure the integrity and completeness of the dataset. Figure 1 illustrates a flowchart summarizing the methodology used to construct the railway network dataset based on train operation records. The process is detailed as follows:

### Step 1. Source data construction

The raw dataset includes multiple data sources: train operation information, geographic coordinates of train stations, train scheduling information, environmental variables, and national holiday records.

#### Train operation records collection

The train operational records include both historical timetables and actual running information for high-speed trains. These data were collected by *TrainStats* (https://trainstats.altervista.org/), a service that downloads daily records from *ViaggiaTreno*, a website operated by the Italian railway company providing real-time delay information. The data are stored in JSON format, with each file representing one day of operations. Each JSON file contains information such as train category, train number, origin and destination stations, scheduled arrival and departure times at each station, delay durations, and general scheduling data for trains on that day. We obtained operational data for the period from 1 January 2024 to 30 June 2024.

#### Railway station geolocation data collection

Geospatial information for Italian railway stations was obtained by extracting the latitude and longitude coordinates associated with each station. A mapping dictionary linking geographic coordinates to station names was subsequently constructed to support spatial referencing and regional analysis. Regional boundary data for Italy were obtained from the GeoJSON Italy repository. By aligning geographic coordinates with regional code definitions, each station was assigned to its corresponding administrative region. The regional classification used in this study is presented in Table 1.

**Table 1:** Mapping between region IDs and administrative regions.

Region Id	Region
01	Piedmont
02	Aosta Valley
03	Lombardy
04	Trentino-Alto Adige/South Tyrol
05	Veneto
06	Friuli Venezia Giulia
07	Liguria
08	Emilia-Romagna
09	Tuscany
10	Umbria
11	Marche
12	Lazio
13	Abruzzo
14	Molise
15	Campania
16	Apulia
17	Basilicata
18	Calabria
19	Sicily
20	Sardinia

#### Major holidays’ data collection

Passenger flow is one of the key factors affecting train operations and is closely related to station dwell time. When passenger volume is high, the extended boarding and alighting time prolongs the station dwell time, thereby raising the likelihood of train delays. However, accurately obtaining the actual number of passengers boarding and alighting at specific stations remains an unresolved challenge^20^. Notably, during major holidays such as New Year’s Day and Easter, station crowd levels are significantly higher than usual. Holiday periods can thus serve as an indicator for assessing the impact of passenger flow on train operations. From January 1 to 30 June 2024, the major holidays considered include: New Year’s Day (1 January 2024), Epiphany (6 January 2024), Easter (20 April 2024), Easter Monday (21 April 2024), Liberation Day (25 April 2024), Labor Day (1 May 2024), Republic Day (2 June 2024).

#### Weather data collection

Weather conditions represent a critical external factor influencing train operations. In this study, temperature, wind, and general meteorological conditions are considered essential variables for analyzing operational performance^33^. Historical weather data spanning January to June 2024 were obtained from the Open-Meteo platform. Data extraction was conducted using the geographical coordinates and corresponding time information of each train station. This weather dataset provides a foundation for evaluating the impact of meteorological variability on railway service reliability and punctuality.

### Step 2. Data curation and correction

In this step, we standardized the data fields and performed consistency checks to correct errors in the collected train operational records. To facilitate large-scale data processing and ensure compliance with standard formats, we used Apache Spark to map the original fields to the GTFS (General Transit Feed Specification) format. The data were then converted from JSON to a more processable CSV format. The raw data contained some duplicate, missing, or erroneous records, which were identified and corrected during preprocessing. Some records contained duplicates, missing values, or inconsistencies, which could lead to inaccuracies when calculating newly derived attributes such as train running time and dwell time. Duplicate records were addressed by identifying discrepancies and removing implausible entries. Missing data were imputed using the most similar operational records, either from adjacent dates with normal operations or from other records on the same day. This approach assumes that a train’s operational pattern remains relatively stable over a short time period.

Additional errors were identified during the calculation of dwell time. For example, some records showed a departure time earlier than the corresponding arrival time, resulting in negative dwell durations, which is not possible in actual train operations. To correct such errors, the average dwell time at the affected station over a recent time window was used as a reference. The corrected dwell time was then added to the arrival time to generate a reasonable and accurate departure time. As a result, we obtained train operational data spanning from 1 January 2024 to 30 June 2024. The dataset covers a 26-week period and includes 2,677,973 operational records from 3,324 trains across seven different service types.

### Step 3. The calculation of actual arrival time, actual departure time, delay time, running time, and dwell time

In this step, we calculated the actual arrival time, actual departure time, delay time, and dwell time of the train using the train operation data.

#### Calculation of actual arrival and departure times

The original dataset does not directly provide the actual arrival and departure times of trains. However, such information is essential for understanding real-world train operations and is a critical component of railway traffic management. We estimated the actual arrival time of each train by adding the reported arrival delay to the scheduled arrival time, and similarly, we computed the actual departure time by adding the departure delay to the scheduled departure time.

#### Calculation of train delay time

Trains typically operate along railway lines according to a fixed timetable, serving a series of stations during each journey. A train’s route includes a starting station ${S}_{1}$, a terminal station ${S}_{k}$, and several intermediate stations ${s}_{i}$ (where $i=2,3,\ldots k-1$). Train delays are generally defined as the difference between the actual time $t$ and the scheduled time $\hat{t}$.

If the difference between the actual and scheduled departure times at station ${S}_{i}$, denoted as ${t}_{d}^{{s}_{i}}-{\hat{t}}_{d}^{{s}_{i}}$, is greater than 0, it is considered a departure delay at that station, represented as ${{DD}}_{i}$. Here, ${t}_{d}^{{s}_{i}}$ is the actual departure time, and ${\hat{t}}_{d}^{{s}_{i}}$ is the scheduled departure time. Similarly, if the arrival time difference at station ${S}_{i}$, expressed as ${t}_{a}^{{s}_{i}}-{\hat{t}}_{a}^{{s}_{i}}$, is greater than 0, it is considered an arrival delay, represented as ${{AD}}_{i}$. In this case, ${t}_{a}^{{s}_{i}}$ is the actual arrival time and ${\hat{t}}_{a}^{{s}_{i}}$ is the scheduled arrival time.

If ${{DD}}_{i}$ or ${{AD}}_{i}$ is less than 0, it indicates that the train departed or arrived earlier than scheduled. If the value is equal to 0, it indicates the train was on time, with no delay. During operation, trains are often affected by various internal or external factors that disrupt their schedules and cause delays. However, the probability of a delay is generally lower than the probability of a train running on time. It is important to note that arrival delays are not applicable at the starting station, and departure delays are not applicable at the terminal station. In the dataset, such values are marked as ‘${\rm{N}}$’.

#### Calculation of train running and dwell time

In a railway transportation system, the operation of a train is composed of two fundamental components: running time and dwell time. Both are critical for evaluating the efficiency of train operations, enhancing passenger experience, and maintaining the overall stability of the transportation system. Running time refers to the time required for a train to travel between two consecutive stations. It is calculated as: ${{rt}}_{i}={t}_{a}^{{s}_{i}}-{t}_{d}^{{s}_{i-1}}$, where ${t}_{a}^{{s}_{i}}$ is the actual arrival time at the current station $c$, and ${t}_{d}^{{s}_{i-1}}$ is the actual departure time from the previous station ${s}_{i-1}$. Shorter and more consistent running times are essential for maintaining schedule adherence and minimizing the risk of delays. Dwell time refers to the duration a train remains at a station, allowing passengers to board and alight. It is calculated as: ${{st}}_{i}={t}_{d}^{{s}_{i}}-{t}_{a}^{{s}_{i}}$, where ${t}_{d}^{{s}_{i}}$ is the actual departure time and ${t}_{a}^{{s}_{i}}$ is the actual arrival time at station ${s}_{i}$. Adequate dwell time is crucial, particularly at major hub stations, to accommodate passenger flow efficiently and to prevent platform congestion and cascading delays. It is worth noting that high-speed trains typically exhibit shorter running and dwell times, which not only ensure adherence to tight schedules but also enhance travel comfort by minimizing station stops and transfer durations.

### Step 4. Adjacent stations mileage computation

Adjacent stations are defined as two stations that are directly connected and appear sequentially along the same railway line. Analyzing the distances, speed limits, and running times between adjacent stations provides valuable insights for optimizing train scheduling. Furthermore, understanding the relationships between adjacent stations aids in designing more efficient passenger routes, improving travel convenience.

To facilitate this analysis, the train operation data were deduplicated, resulting in the identification of 1,290 unique train routes. We obtained the actual inter-station distances of the Italian railway network from the official website of Rete Ferroviaria Italiana (RFI) (https://normativaesercizio.rfi.it/NormativaEsercizio/) and manually entered the corresponding distances for each station into the route file. For a few pairs of stations where the official distance data were unavailable, the distances between adjacent stations were calculated using the geodesic function from the *geopy* library, based on their latitude and longitude information. This function computes the shortest path over the Earth’s surface according to the Haversine formula, expressed as:

1 $$d=2r\cdot \arcsin \left(\sqrt{{\sin }^{2}\left(\frac{\Delta \phi }{2}\right)+\cos \left({\phi }_{1}\right)\cos \left({\phi }_{2}\right){\sin }^{2}\left(\frac{\Delta \lambda }{2}\right)}\right)$$

where *d* represents the distance between the two geographic points, *r* is the radius of the Earth, ${\phi }_{1}$ and ${\phi }_{2}$ are the latitudes of the two stations, $\Delta \phi$ is the difference in latitudes, and $\Delta \lambda$ is the difference in longitudes. By applying this method, inter-station distances were obtained, serving as a critical basis for further analyses of train travel time, speed profiles, and schedule optimization. It should be noted that these inter-station distances, derived from the Haversine formula, differ from the actual operational railway mileage.

### Step 5. Statistical analysis and visualization of delays at train hub stations

**Fig. 2:**
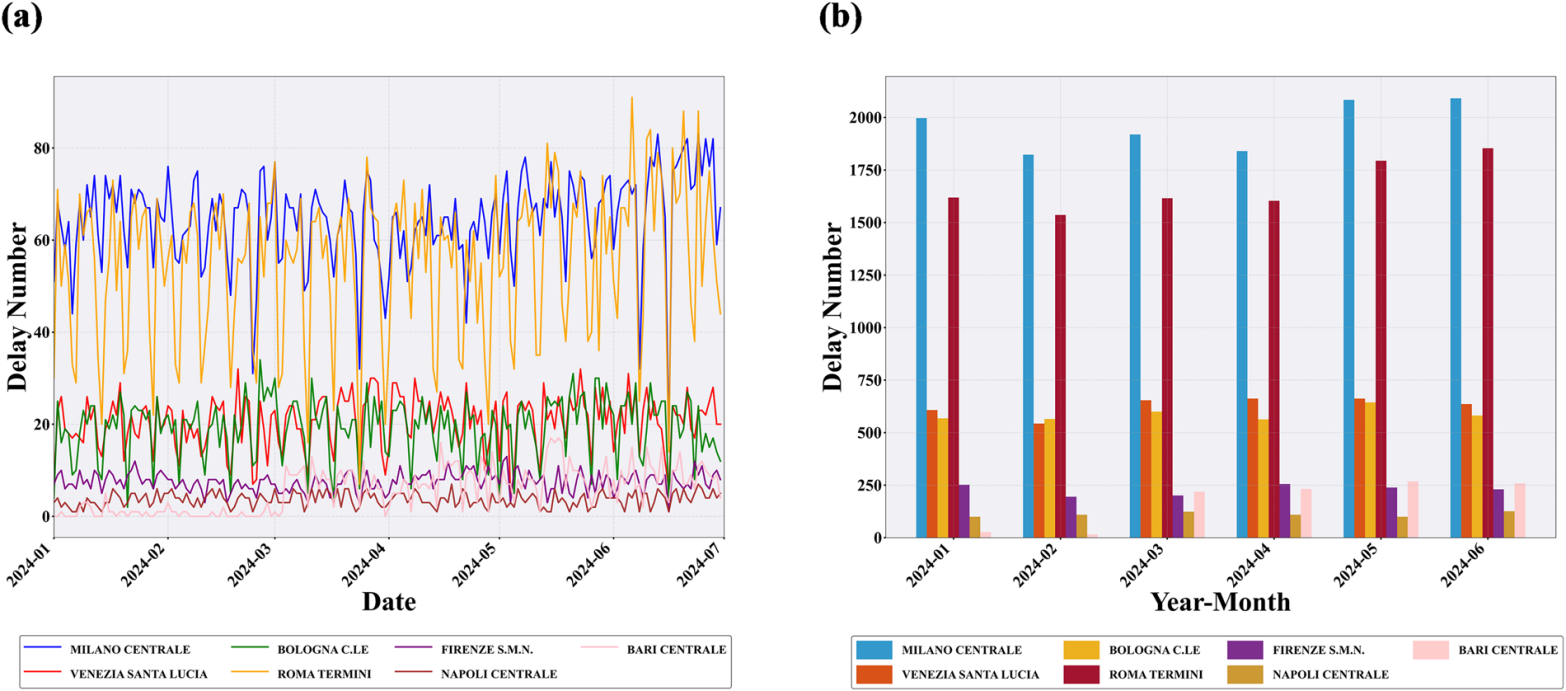
Delay distribution map of hub stations. (**a**) Trend chart of showing delays at hub stations. (**b**) Cumulative bar chart of delays at hub stations.

In this step, the analysis focused on the spatial distribution and frequency of train delays in Italy. To facilitate regional comparison, the country was geographically segmented into three regions: Northern Italy, Central Italy, and Southern Italy. The number of delay events occurring at hub stations within each region was calculated. The list of hub stations analyzed is shown in Table 2. Two types of visualizations were employed to represent the delay data. A line chart depicts the temporal trends at these hub stations. A bar chart shows the cumulative number of delays per station. These visual representations, shown in Fig. 2, provide an intuitive understanding of delay patterns at key operational nodes within the railway network.

**Table 2:** Junction stations.

Area	Station Name
Northern	Milano Centrale
	Venezia Santa Lucia
	Bologna Centrale
Central	Roma Termini
	Firenze Santa Maria Novella
Southern	Napoli Centrale
	Bari Centrale

### Step 6. Analysis of the complexity of railway network topology

The Italian railway system represents one of Europe’s most important transport networks, consisting of intercity, regional, and high-speed services, as summarized in Table 3. IC (InterCity) trains connect major cities, offering medium-speed services suitable for short-distance to medium-distance travel. ICN (InterCity Notte) trains operate overnight, providing sleeper or seating accommodations for long-distance travel. REG (Regionale) trains serve local and regional travel needs, connecting cities with surrounding towns at lower speeds with frequent stops. EC (EuroCity) trains facilitate international travel by linking Italy with major cities in neighboring European countries. FA (FrecciaArgento) Silver Arrow trains deliver high-speed services for medium-distance to short-distance routes, while FB (FrecciaBianca) White Arrow trains provide domestic services with speeds and service levels between regional and high-speed trains. FR (Frecciarossa) Red Arrow trains are the fastest in Italy, operating on dedicated high-speed lines at speeds exceeding 300 km/h, offering a highly comfortable travel experience.

**Table 3:** Train categories.

Train Category	Train Name
IC	InterCity
ICN	InterCity Notte
REG	Regionale
EC	EuroCity
FA	FrecciaArgento
FB	FrecciaBianca
FR	Frecciarossa

**Fig. 3:**
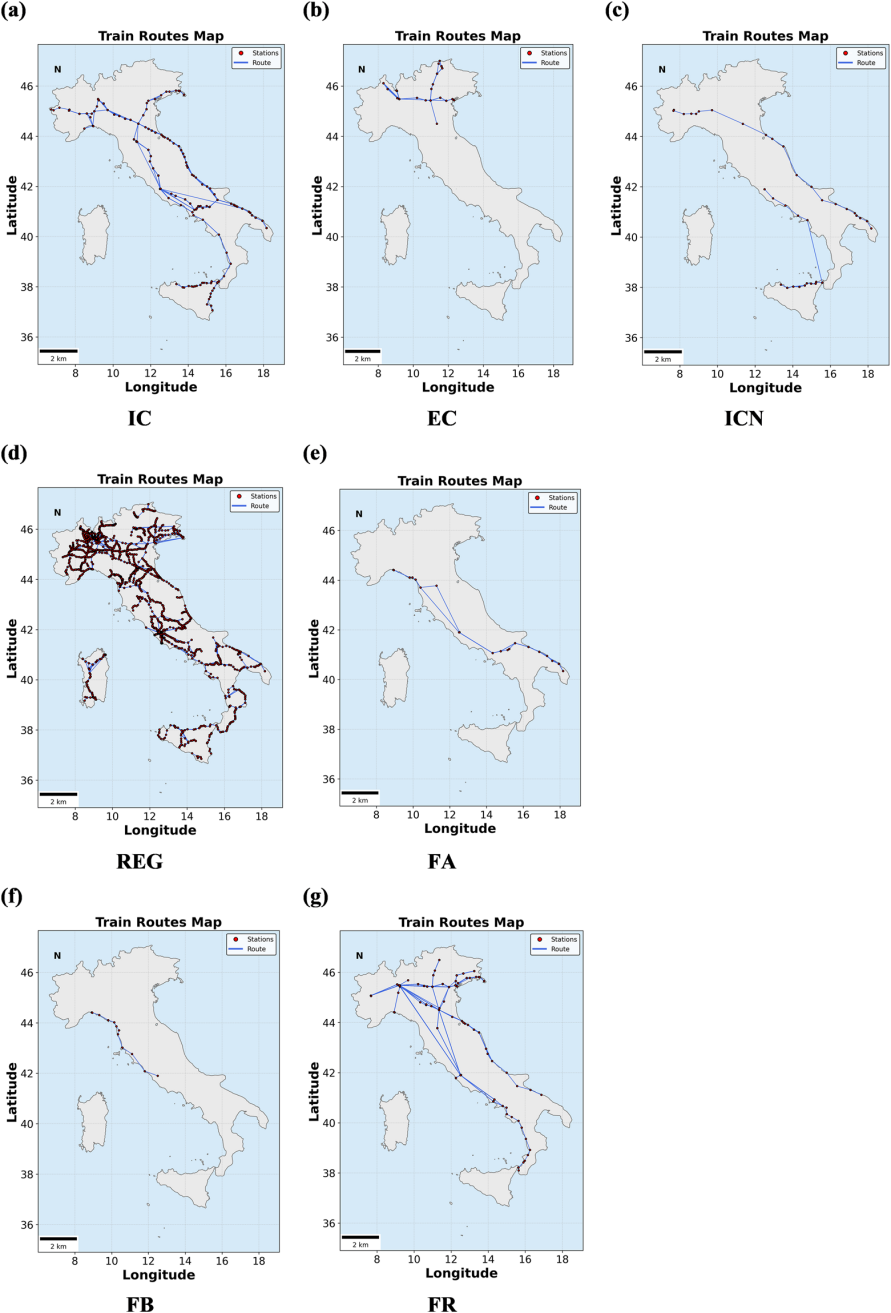
Train routes.

**Fig. 4:**
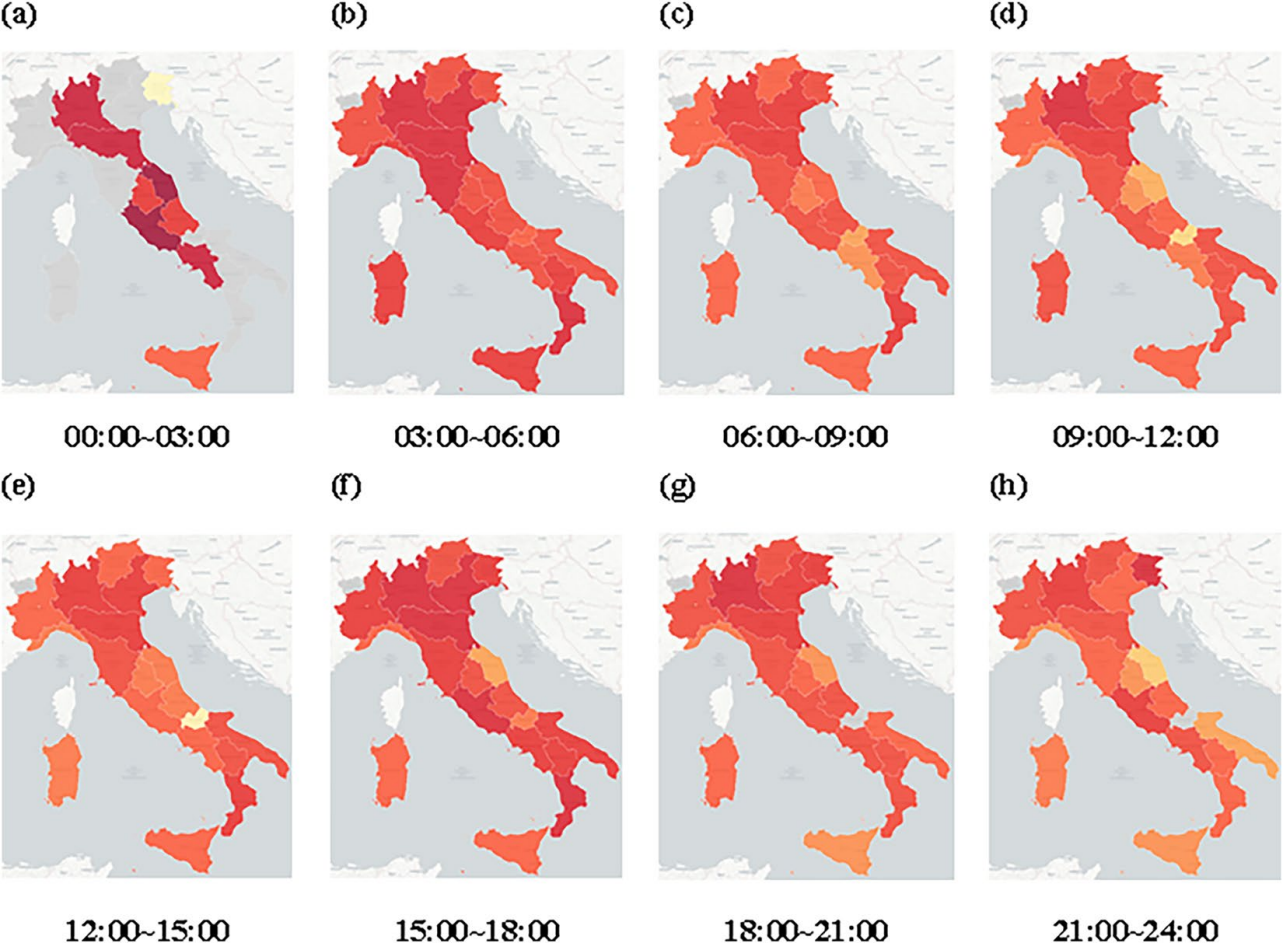
Region delay heat matrices.

These train categories show distinct spatial and temporal operation patterns, influenced by factors such as population density, topographical features, and regional economic activities. As illustrated in Figs. 3, 4, northern Italy, characterized by a denser railway network and more frequent services, experiences more pronounced delays, particularly in metropolitan areas such as Milan, Turin, and Venice. In contrast, southern regions, with relatively fewer trains and lower operational speeds, encounter fewer delays. Furthermore, the western railway corridor along with Tyrrhenian Sea demonstrates higher operational density and service levels compared to the eastern corridor along with Adriatic Sea, where reliance on intercity and regional services results in relatively lower frequencies and fewer delays.

### Step 7. Addition of holiday, cross-day information, weather conditions and adjustments to train scheduling information

In this step, supplementary contextual information including public holidays, cross-day operation indicators, weather conditions, and scheduling adjustments was integrated into the processed train operation dataset to enhance its richness and analytical utility.

#### Addition of major holiday information

Key national holidays in Italy during the data collection period were identified and annotated within the dataset. These include January 1, 2024 (New Year’s Day), January 6, 2024 (Epiphany), April 20, 2024 (Easter), April 21, 2024 (Easter Monday), April 25, 2024 (Liberation Day), May 1, 2024 (Labor Day), and June 2, 2024 (Republic Day). A new attribute labeled “holiday” was added to each train operation record. This attribute is represented as a Boolean value (True or False) to indicate whether the operation date coincides with any of the listed national holidays.

#### Addition of cross-day operation information

In the dataset of train operations, certain trains span across multiple days due to factors such as long-distance routes, nighttime services, or specific scheduling constraints. To maintain the integrity and accuracy of the dataset, an additional attribute labeled “next_day” was introduced. When the “next_day” attribute is set as True, it indicates that the train’s operation extended beyond a single day. Conversely, if the “next_day” attribute is set to False, it indicates that the train’s operations were confined to a single day.

#### Integration of weather-related data

This study incorporates meteorological data, including temperature and general weather conditions (such as heavy snow, hail, etc), for 2,954 train stations across Italy, spanning the period from 1 January to 30 June 2024. To assess the influence of external environmental factors on train operations, the weather information was aligned and merged with the train operation records based on corresponding station names and dates. Strong wind conditions can significantly impact train operations, as trains typically reduce their operating speed under high wind circumstances, affecting both delays and scheduling. To account for this factor, wind-related data have been integrated into our dataset.

#### Adjustments of train dispatching information

Train dispatch information plays a vital role in optimizing railway operations, especially when dealing with disruptions caused by natural disasters or infrastructure maintenance. Extreme weather events such as strong winds and heavy rainfall, along with planned equipment servicing, may result in train suspensions or rerouted services to mitigate operational impacts. By analyzing dispatch information, particularly adjustments involving train cancellations and route changes, railway authorities can gain a comprehensive understanding of train operations and make informed, intelligent dispatching decisions, thereby improving system resilience and efficiency^5^. The dispatch records include cases where trains were rerouted with temporary stops added, rerouted with certain scheduled stops canceled, rerouted with both cancellations and additional temporary stops, and instances where the departure or final destination station was changed. To facilitate interpretation, all original Italian dispatch descriptions were translated into English.

### Step 8. Acquisition of train delay cause information

The railway system constitutes a highly interconnected dynamic network, in which the delay of a single train can propagate through a “domino effect,” affecting trains across multiple subsequent lines and stations. Identifying the causes of delays is crucial for accurately tracing their sources and mitigating the risk of delay propagation. The TrainStats platform retains real-time alert logs for only one year; these logs record instantaneous delay triggers (e.g., signal failures, track maintenance, and power outages), along with corresponding timestamps and location labels. To provide example data, we collected logs from September 20 to September 30, 2024, using a web crawler. We also provide the corresponding code so that researchers can obtain train delay cause information for specific time periods as needed.

### Step 9. Data validation

To ensure the reliability and integrity of the dataset, a comprehensive validation process was conducted, examining train operational records, data distributions, holiday annotations, train scheduling updates, and external environmental factors. Detailed descriptions of the validation procedures are provided in the corresponding data validation section.

## Data Records

### Data records description

The dataset^34^ is publicly available on Figshare and is provided in four separate CSV files, described as follows:

1. **Train operation data.csv**: This file contains operational data for 3324 trains recorded between 1 January 2024 and 30 June 2024, including major holiday indicators and information on train scheduling adjustments.
2. **Train station locations data.csv**: This file provides the latitude, longitude, and regional information for 2,974 train stations across Italy.
3. **Adjacent railway stations mileage data.csv**: This file records the mileage between adjacent stations across 1,290 train routes.
4. **Train fault information.csv**: This file records the duration of train arrival delays and categorizes the primary causes of such delays.

Detailed descriptions of the fields contained in these files are provided in Tables 4–7.

**Table 4:** Train operation station data structure.

Column	Data Type	Description
train_class	String	Category of the trains (e.g., intercity, regional, high-speed).
train_number	Integer	A unique number is assigned to each train.
first_station_name	String	Name of the train’s starting station.
final_station_name	String	Name of the train’s terminal station.
scheduled_departure_station	String	Scheduled departure station of the train.
scheduled_arrival_station	String	Scheduled terminal station of the train.
fault_describe	String	Trains dispatch information, describing operational adjustments or disruptions.
initial_delay	Integer	Delay time at the starting station. (minutes).
final_delay	Integer	Delay time at the terminal station. (minutes).
date	Date	Operating date of the train.
train_id	Integer	Unique identifier for each train instance.
station_name	String	Name of the stations along the train’s route.
arrival_delay	Integer	Delay upon arrival time at each station (minutes).
departure_delay	Integer	Delay upon departure time from each station (minutes).
scheduled_arrival_time	Date	Scheduled arrival time at each station.
scheduled_departure_time	Date	Scheduled departure time from each station.
next_day	Boolean	Indicates whether the train operates across calendar days (“True” for overnight operations).
station_order	Integer	The sequential order of stations along the train’s route.
scheduled_running_time	Integer	Scheduled running time between two consecutive stations (minutes).
scheduled_stop_time	Integer	Scheduled stop duration at each station (minutes).
actual_arrival_time	Date	Actual arrival time recorded at each station.
actual_departure_time	Date	Actual departure time recorded at each station.
actual_running_time	Integer	Actual running time between two consecutive stations (minutes).
actual_stop_time	Integer	Actual stop duration at each station (minutes).
week	Integer	Day of the week (1 = Monday,…, 7 = Sunday).
holiday	Boolean	Indicates whether the operating date coincides with a major public holiday (“True” or “False”).
temperature_min	Float	Minimum temperature (°C) recorded on the operating day at the location of the station.
temperature_max	Float	Maximum temperature (°C) recorded on the operating day at the location of the station.
weather	String	General weather conditions at the location of the station on the operating day.
wind	Float	Maximum wind speed (m/s) recorded on the operating day at the location of the station.

**Table 5:** Train station location data.

Column	Data Type	Description
name	String	Full name of the railway stations.
station_id	Integer	A unique identifier is assigned to each station.
name_short	String	Abbreviated or short form of the station name.
lat	Float	Geographic latitude of the station.
lon	Float	Geographic longitude of the station.
id_region	Integer	Code representing the Geographical region where the station is located.

**Table 6:** Mileage data of adjacent stations.

Column	Data Type	Description
train_id	String	Unique identifier for each train.
route_id	Integer	Identifier for the route taken by the train.
station_name	String	Name of the railway station through which the train passes.
station_order	Integer	Sequential order of the station along the train’s route.
lat	Float	Latitude coordinate of the station.
lon	Float	Longitude coordinate of the station.
distance	Float	Distance (in kilometers) between a departure station and an arrival station.

**Table 7:** Description of fields in Train fault information.

Column	Data Type	Description
date	Date	Date of the recorded train delay.
line	String	The specific line segment where the train delay occurred.
delay_reason	String	The causal factor of the train delay.
delay_duration	Float	The duration of the train delay (in minutes).

## Technical Validation

This section aims to assess whether the train operation records in the railway network dataset accurately reflect actual train operational conditions. A comprehensive validation was conducted based on numerical comparisons and domain-specific analysis, focusing on the following threeaspects:

1. **Accuracy of train operation schedule**: Verifying the consistency between recorded and actual schedules.
2. **Distribution characteristics of train operations**: Verifying operational patterns such as delays, frequency, and spatial-temporal distributions.
3. **Correlation between train operations and external environmental factors**: Examining the relationships between train operations and external influences such as weather conditions and holidays.

### Verification of the train timetable

To verify the accuracy of the scheduled arrival and departure times recorded in the train operation dataset, we obtained the official Italian railway timetables for 2024 from the website (https://www.lcartello.it/Orari-pdf/2023_12_Trenitalia.pdf). The timetable information was utilized to cross-validate the consistency and reliability of the recorded train operation data.

### Verification of train operation distribution characteristics

**Fig. 5:**
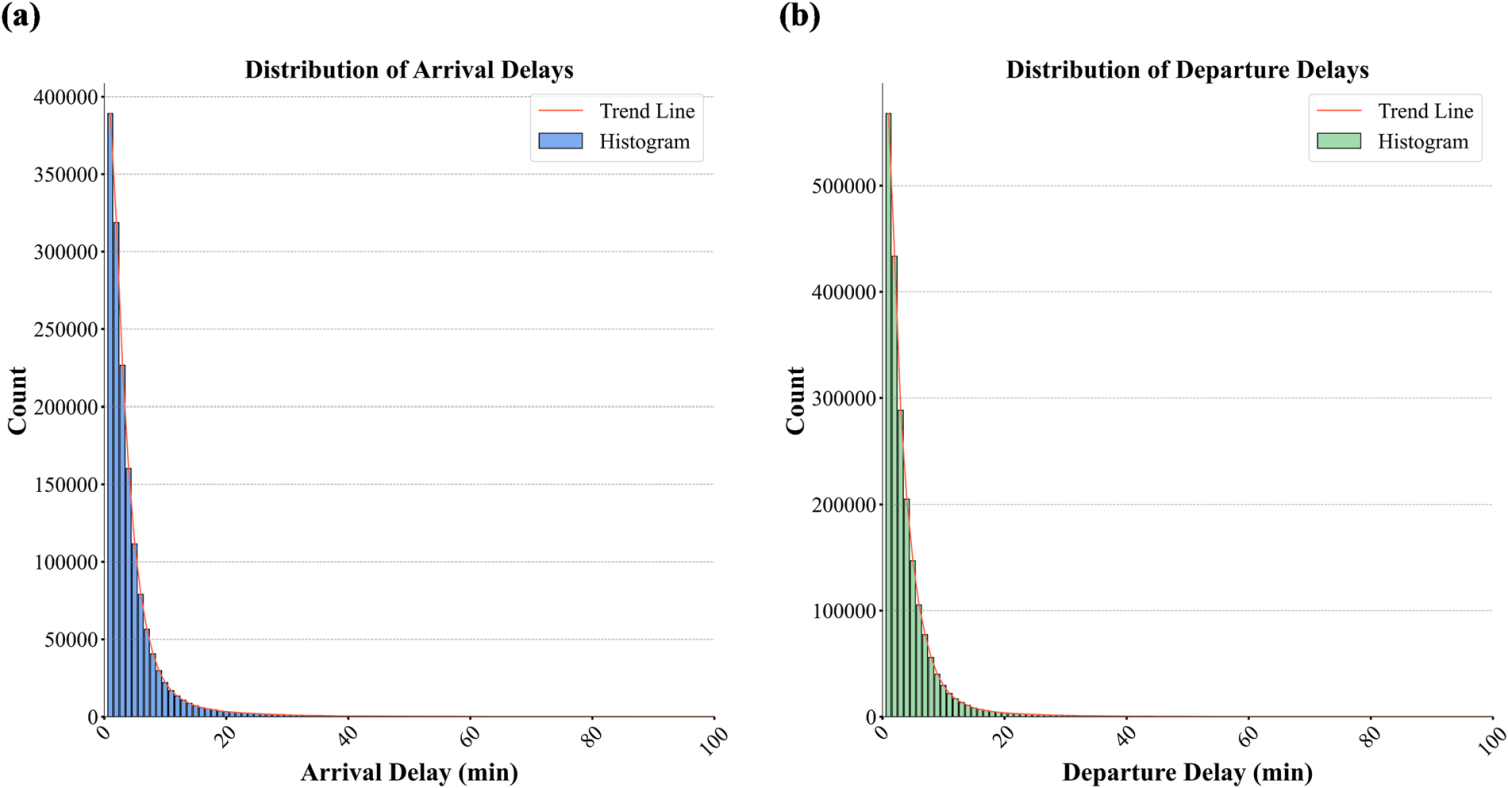
Delay distribution. (**a**) Distribution of train arrival delays. (**b**) Distribution of train departure delays.

**Fig. 6:**
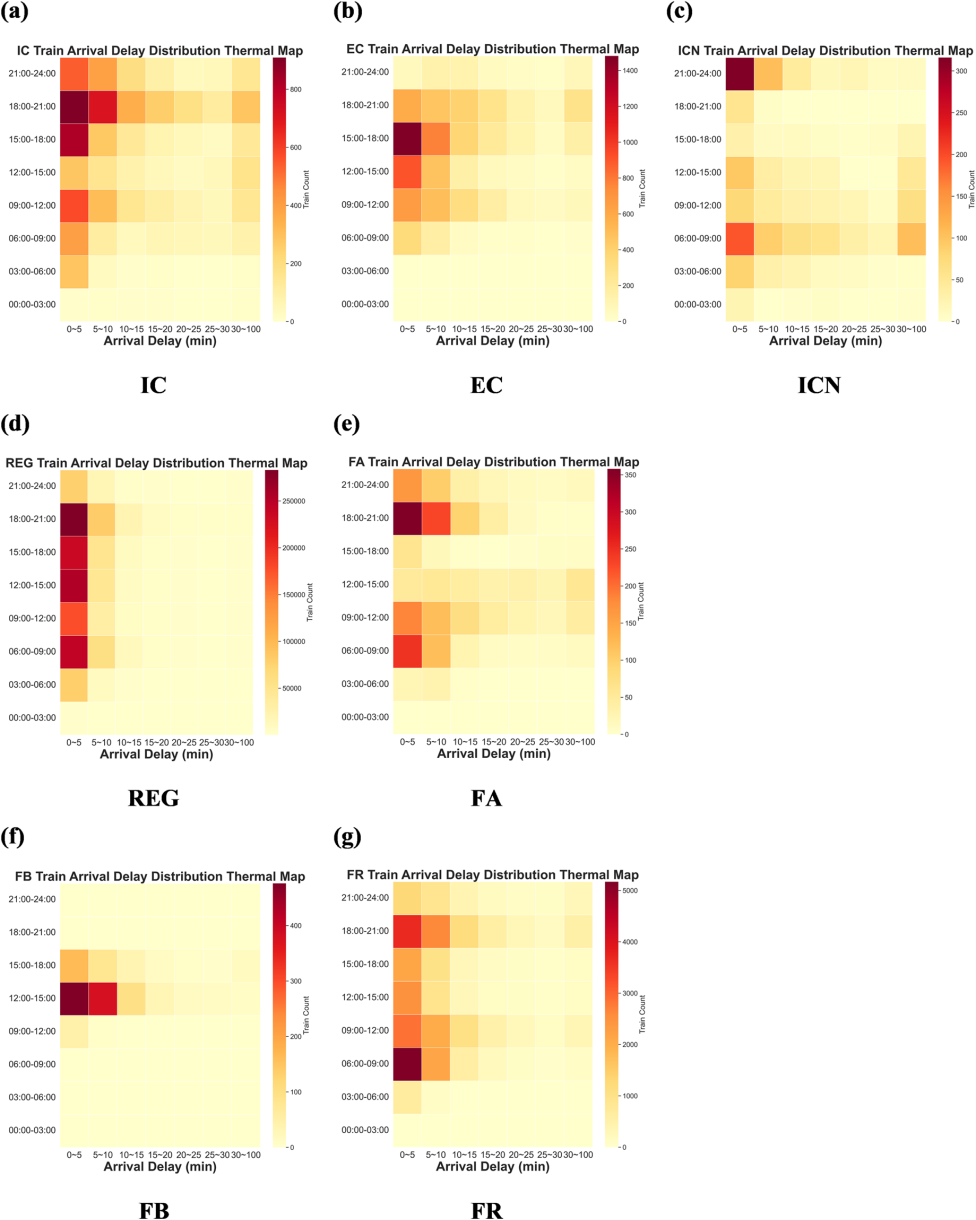
Heatmap illustrating the daily delay patterns for the seven categories of trains.

The verification of train operation distribution characteristics primarily examines delay patterns, running time, and dwell time distribution. As illustrated in Fig. 5, both arrival and departure delays exhibit a long-tailed distribution and demonstrate a linear relationship, indicating a strong correlation between station arrival and departure delays. This observed pattern is consistent with delay distributions reported in other railway networks, where extended delays occur less frequently^18,35^. Furthermore, as discussed in [34], train punctuality is often influenced by the local train load factor, with punctuality decreasing during peak weekday hours due to higher traffic density and improving during off-peak and non-working periods. Figure 6 presents a 24-hour heatmap of train delays, highlighting that IC, EC, REG, FA, FB, and FR trains experience more severe delays during peak periods, while ICN trains encounter significant delays during nighttime operations owing to their overnight service nature.

**Fig. 7:**
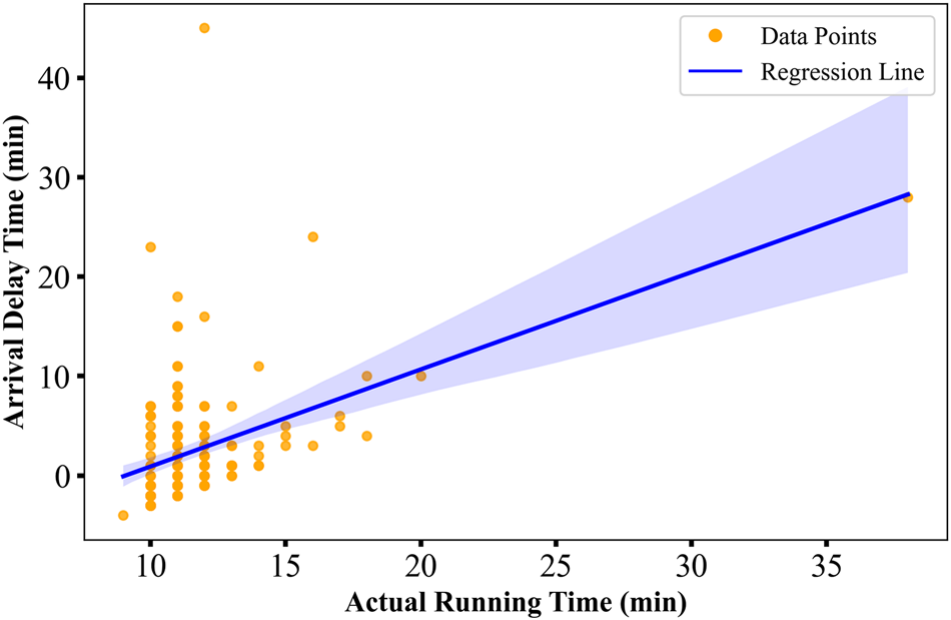
Correlation between arrival delays at Novara station and the actual running time between Vercelli and Novara.

Actual dwell time and travel time are two crucial factors influencing train delays, both exhibiting dynamic correlations with operational punctuality. To examine the relationship between travel time and delays, the actual travel time between Vercelli and Novara stations was analyzed. As shown in Fig. 7, the vertical axis represents the arrival delay at Novara station, while the horizontal axis indicates the actual running time from Vercelli to Novara. The fitted curve clearly shows that longer travel times are associated with greater arrival delays, consistent with general train operation dynamics.

**Fig. 8:**
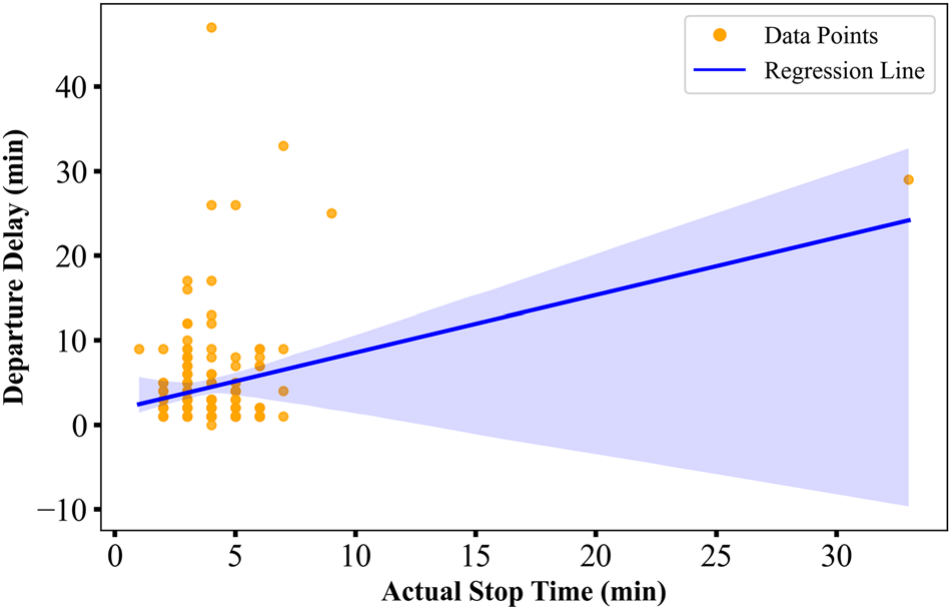
Correlation between actual dwell time at Novara station and the delayed departure time from Novara station.

Similarly, to investigate the relationship between actual dwell time and departure delays, the dwell time at Novara station was analyzed. As depicted in Fig. 8, the fitted curve indicates that extended dwell times are positively correlated with more severe departure delays.

### Verification of the correlation between train operations and external environmental factors

**Fig. 9:**
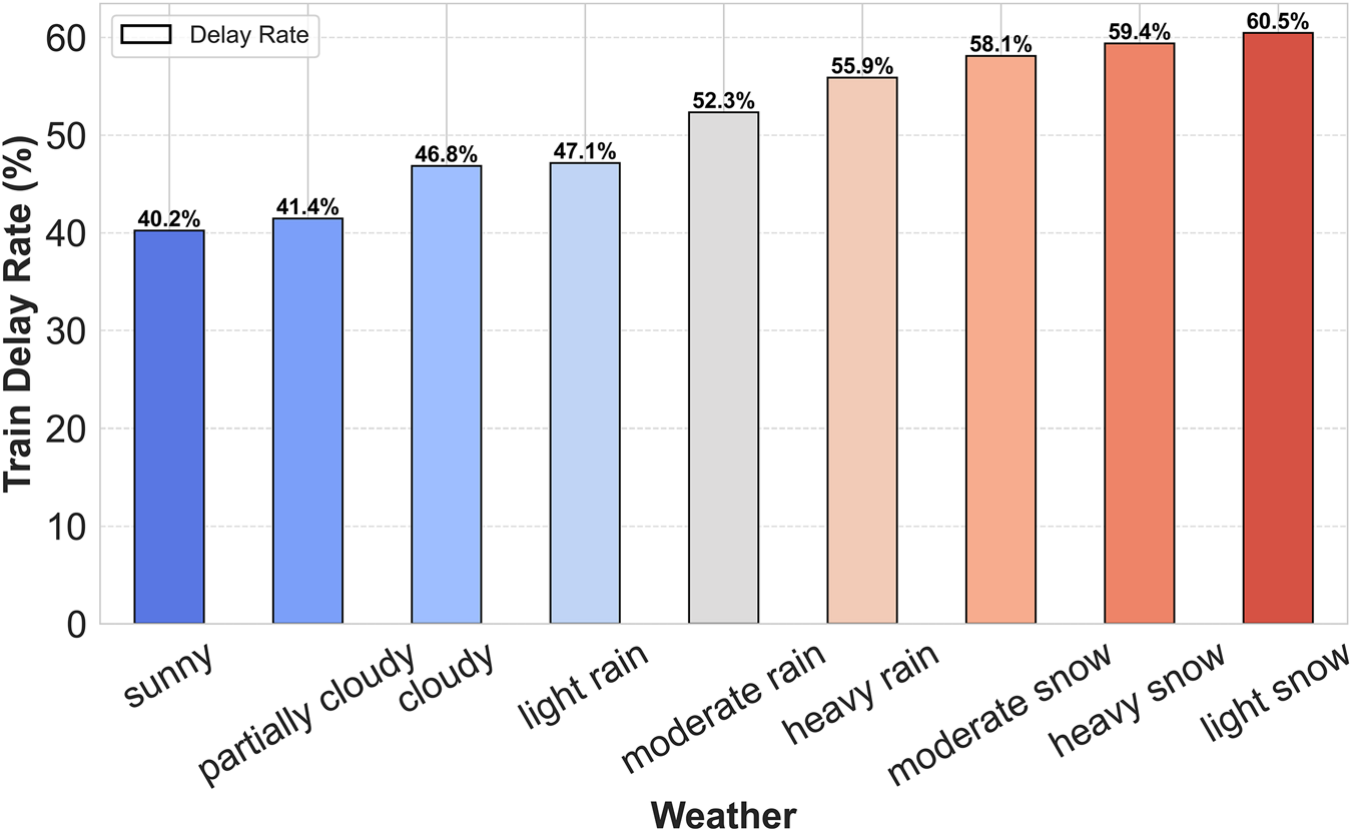
Relationship between weather conditions and train delay rates.

External environmental factors, particularly weather conditions, exert a significant impact on train operations by affecting train performance and operational stability. To quantitatively assess the influence of weather on train delays, we analyzed the correlation between train delay rates and various weather conditions. The delay rate was computed using the following formula: ${\rm{Delay\; Rate}}=\left(\frac{{\rm{Number\; of\; Delayed\; Trains}}}{{\rm{Total\; Number\; of\; Trains}}}\right)\times 100 \%$. As illustrated in Fig. 9, adverse weather conditions, including moderate rain, heavy rain, light snow, moderate snow, and heavy snow, are associated with higher delay rates, all exceeding 0.5, indicating a positive correlation with increased delays. In contrast, clear and cloudy conditions correspond to delay rates below 0.5, suggesting a comparatively lower impact. This result corroborates findings reported in Reference^35^, confirming that weather conditions in the dataset significantly affect train operations.

Overall, through verification across the four aspects outlined above, the dataset is demonstrated to be reliable. It offers a robust foundation for research in intelligent transportation systems, data mining and traffic demand forecasting.

## Data Availability

The Italian Railway Network Dataset supporting this Data Descriptor is openly available in Figshare at 10.6084/m9.figshare.28891607.v2. The dataset can be freely used for research and educational purposes.

## Code availability

We share our code for data processing and generation in GitHub (https://github.com/1245642138/train_data_process.git).
